# Case Report: Paradoxical worsening of hypoxemia with PEEP during one-lung ventilation: a case of pressure-dependent intracardiac shunting

**DOI:** 10.3389/fcvm.2026.1878145

**Published:** 2026-06-15

**Authors:** Xinrui Yin, Shijia Du

**Affiliations:** 1 Department of Anesthesiology, Aerospace Center Hospital, Beijing, China; 2 Department of VIP Dental Service, Peking University Stomatological Hospital, Beijing, China

**Keywords:** case report, intracardiac shunt, one-Lung ventilation, patent foramen ovale, positive end-expiratory pressure

## Abstract

**Background:**

Perioperative hypoxemia during one-lung ventilation (OLV) is commonly managed by applying positive end-expiratory pressure (PEEP) to recruit the dependent lung. However, in rare instances, PEEP escalation may paradoxically compromise oxygenation. We describe a case in which a transient rise in right-sided pressures relative to left-sided pressures likely unmasked a pressure-dependent right-to-left intracardiac shunt.

**Case presentation:**

A 55-year-old male underwent elective video-assisted thoracoscopic surgery (VATS) right upper lobe resection. During otherwise stable OLV, mild hypoxemia (SpO_2_ 88%–90%) prompted escalation of PEEP from 5 to 10 cmH_2_O. Paradoxically, SpO_2_ abruptly declined to 82% within 2 min despite hemodynamic stability and no evidence of an acute mechanical or surgical cause. Suspecting a pressure-dependent shunt, PEEP was reduced to 2 cmH_2_O, with rapid recovery of SpO_2_ to 96%–98%. Emergent intraoperative transthoracic echocardiography was nondiagnostic owing to limited acoustic windows. Postoperative evaluation identified an anatomical PFO on transesophageal echocardiography and a small right-to-left shunt on contrast-enhanced transcranial Doppler, supporting the suspected pressure-dependent shunting mechanism.

**Conclusions:**

This case illustrates that PEEP can be a double-edged sword during OLV. Under certain conditions, escalating airway pressure may transiently reverse the interatrial pressure gradient and facilitate right-to-left shunting across a PFO. When oxygenation deteriorates following a recruitment maneuver, clinicians should resist reflexive PEEP escalation and consider an intracardiac shunt as a potentially reversible cause; in this context, PEEP reduction may serve as both a diagnostic probe and a therapeutic rescue.

## Introduction

One-lung ventilation (OLV) is indispensable in thoracic surgery, yet perioperative hypoxemia remains a frequent challenge and often demands rapid decisions under incomplete information. When oxygenation deteriorates during OLV, a common initial response is to optimize ventilation–perfusion matching of the dependent lung, and positive end-expiratory pressure (PEEP) is frequently used as a first-line recruitment strategy (1, 2). Under typical physiological conditions, titrated PEEP recruits atelectatic units, increases functional residual capacity, and improves arterial oxygenation. However, PEEP is not merely a pulmonary maneuver: its effects extend beyond the pulmonary parenchyma, and escalating airway pressure does not universally translate into improved gas exchange (1, 2).

The prevalence of patent foramen ovale (PFO) in the general population is substantial, affecting approximately 25%–30% of adults, although most remain asymptomatic throughout life (3). In the context of thoracic surgery, this prevalence suggests that a meaningful proportion of patients undergoing OLV may harbor an occult PFO. However, routine preoperative screening is not standard practice in asymptomatic patients, and the interaction between positive-pressure ventilation and PFO physiology remains an underappreciated consideration in thoracic anesthesia.

In patients with an occult PFO, PEEP escalation may paradoxically contribute to clinical deterioration by favoring pressure-dependent right-to-left shunting when right-sided pressures rise relative to left-sided pressures (4). This mechanism can be overlooked intraoperatively, particularly when TEE is technically challenging in the setting of double-lumen tube ventilation, lateral positioning, and an evolving intraoperative crisis. We describe a case of severe refractory hypoxemia during OLV in which increasing PEEP consistently worsened oxygenation, whereas reduction of PEEP produced immediate improvement. Postoperative studies confirmed an anatomical PFO on TEE and demonstrated a small right-to-left shunt at rest and after provocation on contrast-enhanced transcranial Doppler, supporting the plausibility of a dynamic shunt during the intraoperative event (5). Although isolated case reports have documented paradoxical responses to PEEP in critically ill patients with PFO, reports in the specific setting of intraoperative OLV during thoracic surgery appear to be very limited. The central lesson is counterintuitive but clinically important: when hypoxemia paradoxically worsens with PEEP escalation, clinicians should consider an intracardiac shunt as a potentially reversible cause—and PEEP reduction may serve as both a diagnostic probe and a therapeutic rescue.

## Case presentation

A 55-year-old male (height: 172 cm; weight: 74 kg; body mass index: 25 kg/m^2^) was scheduled for elective video-assisted thoracoscopic surgery (VATS) right upper lobe resection for a 2.5-cm solid pulmonary nodule. His medical history was unremarkable, with no known cardiac or pulmonary disease, and he denied any prior history of stroke, syncope, or unexplained dyspnea. Preoperative electrocardiography and pulmonary function tests were within normal limits. Given the absence of symptoms or clinical findings suggestive of an intracardiac shunt, preoperative transthoracic echocardiography (TTE) was not performed, consistent with routine practice in asymptomatic patients.

General anesthesia was induced according to standard institutional protocols. A 37-Fr left-sided double-lumen tube (DLT) was inserted, and correct positioning was confirmed by fiberoptic bronchoscopy (FOB) in both the supine and left lateral decubitus positions. Following the initiation of OLV in the left lateral decubitus position, mechanical ventilation was delivered using pressure-regulated volume control (PRVC) mode with the following parameters: tidal volume 450 mL [approximately 6.6 mL/kg predicted body weight (PBW: 67.8 kg)], respiratory rate 14 breaths/min, PEEP 5 cmH_2_O, and FiO_2_ 0.5. Initial oxygenation was excellent, with SpO_2_ maintained at 99%–100%, stable hemodynamics [mean arterial pressure (MAP) approximately 78 mmHg], and peak airway pressure of 18–20 cmH_2_O.

Approximately 20 min after the initiation of OLV, a gradual decline in oxygen saturation was observed, with SpO_2_ decreasing to 88%–90%. During this period, there was no evidence to suggest tension physiology, surgical compression of the ventilated lung, or hemodynamic instability. In response to this evolving hypoxemia, FiO_2_ was increased to 1.0, and immediate fiberoptic bronchoscopy confirmed persistent optimal DLT positioning with no airway obstruction or significant secretions. To address suspected dependent-lung atelectasis, PEEP was escalated from 5 to 10 cmH_2_O, during which MAP remained stable at approximately 75 mmHg without additional vasoactive support.

Paradoxically, within 2 min of escalating PEEP to 10 cmH_2_O, SpO_2_ dropped abruptly to 82% (from a baseline of 88%–90%) (Table 1). This deterioration occurred despite preserved hemodynamic stability, with MAP remaining approximately 75 mmHg. Ventilatory pressures remained within an expected range (peak/plateau airway pressures 29/24 cmH_2_O), and there were no accompanying findings to suggest tension physiology or an acute loss of dependent-lung compliance. The discordant response to PEEP—worsening rather than improvement in oxygenation following a standard recruitment strategy—prompted immediate consideration of a pressure-dependent right-to-left intracardiac shunt.

**Table 1:** Intraoperative physiological trends during hypoxemia troubleshooting.

Time from OLV initiation (min)	Clinical phase	PEEP (cmH_2_O)	FiO_2_	SpO_2_ (%)	Ppeak/Pplat (cmH_2_O)	MAP (mmHg)	Clinical observations and interventions
0	Initial OLV	5	0.5	99–100	19/16	∼78	Baseline stable oxygenation.
∼20	Onset of hypoxemia	5	0.5 → 1.0	88–90	21/18	∼76	FiO_2_ increased; DLT position reconfirmed by FOB; no tension physiology or surgical compression.
∼22	PEEP escalation	5 → 10	1.0	88–90	–	∼75	PEEP increased to treat suspected dependent-lung atelectasis.
∼24	Nadir desaturation	10	1.0	82	29/24	∼74–75	Paradoxical deterioration approximately 2 min after PEEP escalation, with preserved hemodynamics.
∼24	PEEP reduction	10 → 2	1.0	82	–	∼75	PEEP reduced because of suspected pressure-dependent intracardiac shunting.
∼27	Recovery	2	1.0	96–98	18/15	∼77	Rapid recovery within approximately 3 min with other ventilator settings unchanged; intraoperative TTE subsequently nondiagnostic because of limited acoustic windows.

Time is reported as approximate minutes from initiation of one-lung ventilation (OLV). Intervention rows and physiological response rows are listed separately to show the temporal relationship between PEEP adjustment and oxygenation. SpO_2_ values represent the observed range within each clinical phase. OLV, one-lung ventilation; PEEP, positive end-expiratory pressure; FiO_2_, fraction of inspired oxygen; SpO_2_, peripheral oxygen saturation; Ppeak, peak airway pressure; Pplat, plateau airway pressure; MAP, mean arterial pressure; DLT, double-lumen tube; FOB, fiberoptic bronchoscopy; TTE, transthoracic echocardiography.

On suspicion of a pressure-dependent intracardiac shunt, PEEP was promptly reduced to 2 cmH_2_O. Oxygenation improved rapidly, with SpO_2_ recovering to 96%–98% within 3 min while FiO_2_ and other ventilator settings remained unchanged. This immediate and reproducible response was strongly consistent with a pressure-dependent shunt. Following this recovery, an emergent intraoperative TTE was performed via the anterior chest wall to assess for interatrial shunting. However, color Doppler imaging did not demonstrate definitive shunt flow, most likely owing to severely limited acoustic windows in the lateral decubitus position with the left hemithorax exposed. The absence of echocardiographic evidence did not exclude a small, intermittent, or pressure-dependent shunt, particularly in light of the temporal relationship between PEEP changes and oxygenation and the rapid response to PEEP reduction.

**Figure 1:**
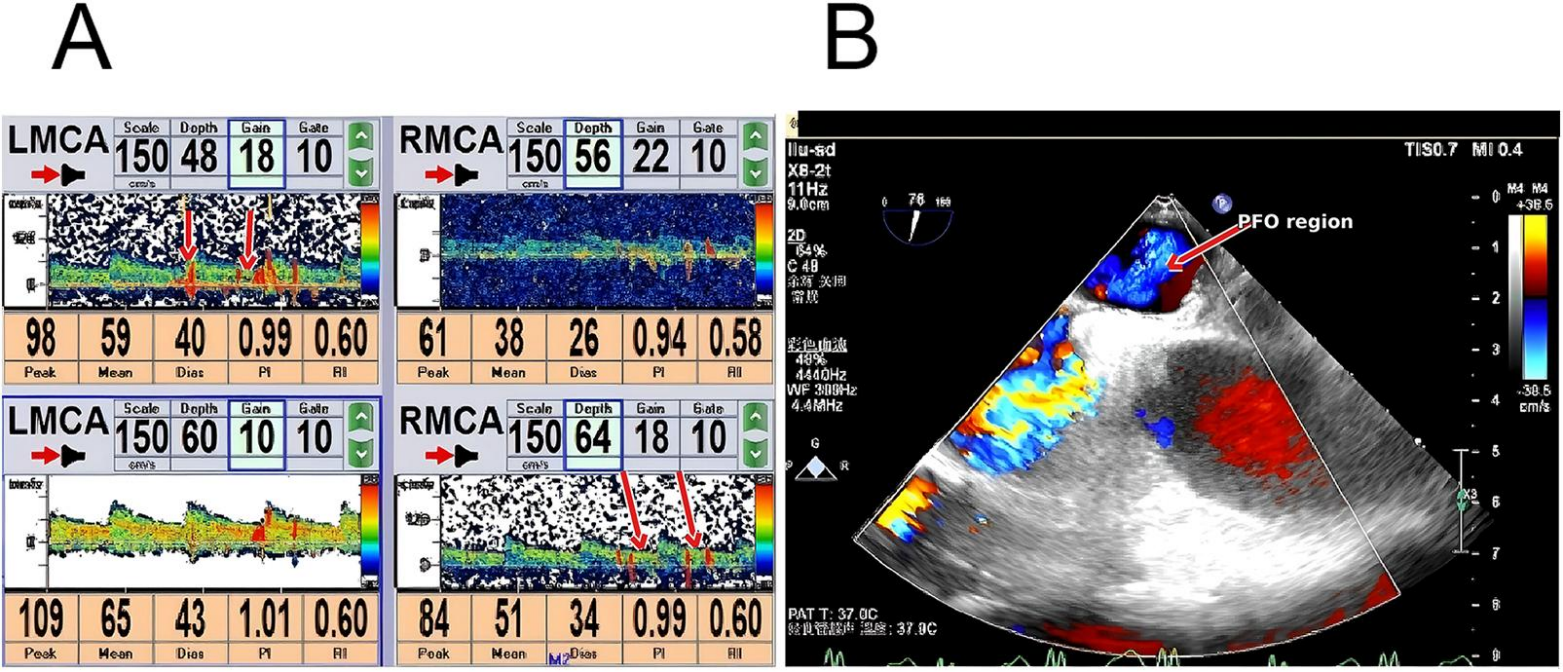
Postoperative imaging findings supporting a patent foramen ovale and a small right-to-left shunt. **(A)** Contrast-enhanced transcranial Doppler demonstrating representative microbubble signals (arrows). **(B)** Transesophageal echocardiography with color Doppler demonstrating interatrial flow at the PFO region (arrow).

Postoperatively, the patient's recovery was uneventful, but the intraoperative event prompted targeted evaluation for an occult shunt. On postoperative day 1, contrast-enhanced transcranial Doppler (c-TCD) was performed as a sensitive, noninvasive screening test. The study demonstrated a positive bubble study with a low microbubble burden (<10 microbubble signals within 10 s, both at rest and after Valsalva maneuver), consistent with a small right-to-left shunt, later anatomically supported by TEE-confirmed PFO (Figure 1A). On postoperative day 2, TEE was performed for anatomical characterization. TEE identified a patent foramen ovale (PFO) with a 2.1-mm opening and a 6.1-mm tunnel length. Notably, under resting postoperative conditions with spontaneous breathing, color Doppler imaging predominantly demonstrated a small left-to-right interatrial shunt, without clear right-to-left flow (Figure 1B).

The contrast between a predominantly left-to-right shunt at rest and the transient right-to-left shunting inferred intraoperatively supports the interpretation that shunt direction was dynamic, likely reversing transiently under intraoperative conditions of elevated right-sided pressures during positive-pressure ventilation with high PEEP. Following multidisciplinary discussion involving anesthesiology, cardiology, and cardiothoracic surgery, the patient was counseled regarding the incidental finding of a PFO. Given the absence of cryptogenic stroke, paradoxical embolism, or decompression illness, and considering that the shunt was small and predominantly left-to-right under resting conditions, conservative management was recommended without routine PFO closure or antiplatelet therapy; the rationale is discussed further below. The patient was advised to avoid activities associated with prolonged Valsalva maneuvers and to inform future anesthesiologists of this finding. He was discharged on postoperative day 5 in good condition, with no recurrence of hypoxemia or other complications, and cardiology follow-up was arranged at 3 months to reassess the need for PFO closure.

## Discussion

This case describes a paradoxical and severe deterioration in oxygenation during OLV following escalation of PEEP. Although PEEP is conventionally applied to recruit atelectatic alveoli in the dependent lung, the response in this patient was the opposite: SpO_2_ fell abruptly from 88%–90% to 82% within 2 min after PEEP was increased from 5 to 10 cmH_2_O, and recovered promptly after PEEP was reduced to 2 cmH_2_O. Subsequent postoperative evaluation identified a PFO with the capacity for pressure-dependent right-to-left shunting, supporting an intracardiac shunt as the most likely mechanism for the intraoperative event. In this setting, a paradoxical response to PEEP may serve as an early physiological clue to a pressure-dependent shunt, highlighting a clinically important pitfall: when oxygenation worsens with recruitment maneuvers, clinicians should reconsider reflexive PEEP escalation and evaluate the possibility of an unrecognized intracardiac shunt (1, 6, 7).

The clinical relevance of this phenomenon is underscored by the high prevalence of PFO, which affects approximately 25%–30% of adults yet remains asymptomatic in most (1, 6). Because routine preoperative echocardiographic screening is not standard in asymptomatic patients, an occult PFO will frequently be unknown at the time of surgery. The intersection of this common anatomical variant with the hemodynamic perturbations of OLV may therefore allow a previously silent PFO to manifest as acute, severe hypoxemia, warranting greater awareness in the thoracic anesthesia community.

Although paradoxical hypoxemia related to PFO has been described in critically ill patients receiving mechanical ventilation, reports specifically documenting this mechanism during intraoperative OLV appear to be very limited. In a focused PubMed search combining terms for patent foramen ovale, right-to-left shunt, one-lung ventilation, PEEP, and perioperative hypoxemia, we found very limited reports specifically focused on intraoperative OLV, lateral positioning, and PEEP-associated PFO shunting. The most comparable published case involved weaning failure from mechanical ventilation due to paradoxical desaturation with PEEP (8); however, that case occurred in an intensive care setting with hemodynamic conditions different from those of our patient. Within this limited literature, the present case adds a detailed account of an acute intraoperative crisis during OLV in which PEEP reduction served as both a diagnostic clue and a therapeutic intervention for an occult PFO-related shunt, with rapid reversibility on PEEP titration and multimodal postoperative characterization of the shunt.

The physiological impact of PEEP during OLV is highly context dependent and reflects a balance between alveolar recruitment and pulmonary vascular–cardiac interactions. In most patients, titrated PEEP improves ventilation–perfusion matching by stabilizing functional residual capacity (7, 8). However, the direction and magnitude of flow across a PFO are governed by the instantaneous pressure gradient between the right and left atria, and several features of OLV may transiently raise right-sided pressures relative to the left. Hypoxic pulmonary vasoconstriction and atelectasis in the non-ventilated lung increase pulmonary vascular resistance; escalation of PEEP in the dependent lung can overdistend compliant alveoli, further increasing pulmonary vascular resistance and right ventricular afterload; and positive-pressure ventilation, lateral positioning, and mediastinal shift alter the transmission of intrathoracic pressure to the cardiac chambers and to systemic venous return (9). The convergence of these factors may briefly reverse the interatrial pressure gradient, opening a right-to-left channel across an otherwise left-to-right or balanced PFO.

This mechanism also explains how an anatomically small shunt can produce a clinically marked desaturation. Blood shunted right-to-left across a PFO is poorly oxygenated venous blood that bypasses pulmonary gas exchange entirely. Because this represents a true intracardiac shunt rather than a ventilation–perfusion mismatch, even a modest shunt fraction can produce a disproportionate fall in arterial oxygen saturation and, unlike V/Q mismatch, responds poorly to increases in FiO_2_. This is consistent with the persistence of hypoxemia in our patient despite an FiO_2_ of 1.0 and with the abrupt yet readily reversible nature of the desaturation. It also reconciles the apparent discrepancy between the intraoperative event and the postoperative findings: the small right-to-left signal on awake c-TCD and the predominantly left-to-right flow on resting TEE reflect baseline conditions, whereas the intraoperative loading was greater than resting postoperative conditions and qualitatively different from brief provocative testing. Resting imaging that shows only a small or predominantly left-to-right shunt therefore does not exclude clinically significant, transient right-to-left shunting under the loading conditions of OLV with high PEEP.

These considerations also underscore that PEEP cannot be applied uniformly across patients. The optimal level is highly individual and is influenced by body habitus, lung and chest-wall compliance, and surgical factors; obese patients, for example, may require higher PEEP levels to counteract elevated pleural and abdominal pressures, whereas the same level could be detrimental in others. Strategies to individualize PEEP—including driving-pressure–guided titration, esophageal (transpulmonary) pressure monitoring, electrical impedance tomography, and decremental PEEP trials—aim to identify the pressure that best balances recruitment against overdistension. The present case adds an important dimension to this individualization: in a patient harboring a pressure-dependent intracardiac shunt, a PEEP level that would be appropriate or even insufficient from a purely pulmonary-mechanical standpoint may nonetheless be harmful by promoting right-to-left shunting. Optimal PEEP selection during OLV should therefore weigh not only lung mechanics but also heart–lung interactions and the possibility of an occult intracardiac communication.

Despite this physiologically coherent explanation, confirming an intracardiac shunt intraoperatively can be diagnostically challenging. In this case, emergent intraoperative TTE did not demonstrate definitive interatrial shunting. Although TTE is noninvasive, its sensitivity for small or pressure-dependent PFO shunts is limited, particularly without agitated saline contrast and provocative maneuvers (10). Intraoperative image quality is further constrained by suboptimal acoustic windows in the lateral decubitus position and by operative limitations. Accordingly, the absence of definitive Doppler evidence intraoperatively should not be interpreted as categorical exclusion of an intracardiac shunt when the physiological evidence—specifically a paradoxical inverse relationship between PEEP and oxygenation—is strong. The suspected shunt mechanism was subsequently supported by postoperative multimodal assessment, including contrast-enhanced transcranial Doppler demonstrating a low microbubble burden (<10 signals within 10 s, both at rest and after Valsalva) consistent with a small right-to-left shunt, and TEE providing anatomical characterization of the PFO (10, 11).

Importantly, paradoxical desaturation with PEEP is not specific to intracardiac shunting, and a pressure-dependent PFO shunt should be considered after common mechanical and pulmonary causes of intraoperative hypoxemia have been addressed. The principal differential diagnoses during OLV, together with their distinguishing features and bedside evaluation, are summarized in Table 2. In our patient, double-lumen tube malposition, airway obstruction or mucus plugging, surgical compression, and tension physiology were excluded before the paradoxical PEEP response was recognized, which is what directed attention toward a pressure-dependent shunt.

**Table 2:** Differential diagnosis of acute hypoxemia during one-lung ventilation.

Cause	Typical features	Bedside evaluation	Initial management
DLT malposition	Sudden desaturation after positioning or surgical manipulation; abnormal capnography; asymmetric breath sounds	Fiberoptic bronchoscopy	Reposition DLT under bronchoscopic guidance
Airway obstruction/mucus plugging	Rising peak airway pressure; reduced tidal volume; secretions	Bronchoscopy; suction	Clear secretions; confirm patency
Dependent-lung atelectasis	Gradual desaturation; low compliance	Auscultation; ventilator waveforms	Recruitment; cautious individualized PEEP titration with close monitoring of oxygenation and hemodynamics
Surgical compression of dependent lung/mediastinal structures	Temporal link to surgical manipulation; hemodynamic change	Communication with surgical team	Release compression; pause manipulation
Impaired hypoxic pulmonary vasoconstriction	Persistent shunt physiology in non-ventilated lung	Diagnosis of exclusion	Optimize FiO_2_; address contributing factors
Pulmonary embolism	Acute desaturation with hemodynamic compromise	EtCO_2_ drop or widened PaCO_2_–EtCO_2_ gradient; TTE/TEE if feasible; hemodynamic assessment	Supportive; specific therapy as indicated
Tension pneumothorax/tension physiology	Hypotension; sudden increase in airway pressure; mediastinal shift or tension physiology observed by the surgical team	Clinical examination; inspection of surgical field	Immediate decompression
Pressure-dependent right-to-left shunt (e.g., PFO)	Paradoxical desaturation with PEEP increase; rapid recovery with PEEP reduction; poor response to FiO_2_	Observe PEEP–oxygenation relationship; postoperative c-TCD/TEE	Reduce PEEP; consider CPAP to non-ventilated lung

Common mechanical, pulmonary, and cardiac causes of acute desaturation during one-lung ventilation, with their characteristic features, bedside evaluation, and initial management. A pressure-dependent right-to-left shunt across a patent foramen ovale should be considered after more common mechanical and pulmonary causes have been addressed, particularly when oxygenation worsens with PEEP escalation and recovers promptly with PEEP reduction. DLT, double-lumen tube; PEEP, positive end-expiratory pressure; FiO_2_, fraction of inspired oxygen; EtCO_2_, end-tidal carbon dioxide; PaCO_2_, arterial carbon dioxide tension; c-TCD, contrast-enhanced transcranial Doppler; TEE, transesophageal echocardiography; PFO, patent foramen ovale.

The primary lesson from this case is that PEEP should not be regarded as a universal remedy for hypoxemia during OLV. Building on the differential diagnosis above, we propose a pragmatic stepwise approach to refractory hypoxemia during OLV, proceeding from mechanical troubleshooting to recognition of a paradoxical PEEP response and postoperative confirmation (Table 3). The central element is Step 4: if oxygenation worsens rather than improves after a cautious PEEP increase, a pressure-dependent intracardiac shunt should be suspected, and reduction of PEEP can serve simultaneously as a diagnostic probe and a therapeutic maneuver, with alternative strategies such as CPAP to the non-ventilated lung considered in parallel.

**Table 3:** Proposed stepwise approach to refractory hypoxemia during one-lung ventilation.

Step	Action	Key points
1. Mechanical troubleshooting	Verify DLT position (auscultation, capnography); confirm circuit integrity and tidal volume delivery	Exclude disconnection, kink, mucus plug
2. Bronchoscopic confirmation	Fiberoptic bronchoscopy to confirm DLT position; suction secretions; communicate with surgical team	Exclude malposition, obstruction, surgical compression
3. Cautious recruitment	Increase FiO_2_ to 1.0; if mechanical causes are excluded, increase PEEP cautiously in small increments, guided by oxygenation, airway pressures, compliance, and hemodynamics	Observe the oxygenation response within ∼2 min
4. Recognize paradoxical response	If SpO_2_ worsens with a PEEP increase, suspect a pressure-dependent intracardiac shunt and return to the last effective/lower PEEP level (in this case, 2 cmH_2_O)	Rapid improvement after PEEP reduction supports the suspicion of a pressure-dependent shunt; PEEP reduction may be both diagnostic and therapeutic; consider CPAP to the non-ventilated lung or intermittent two-lung ventilation
5. Postoperative confirmation	Contrast-enhanced transcranial Doppler as a sensitive screen; TEE for anatomical characterization; cardiology consultation	Counsel patient; document for future anesthetic management

A pragmatic five-step approach to refractory hypoxemia during one-lung ventilation, proceeding from mechanical troubleshooting to recognition of a paradoxical PEEP response and postoperative confirmation. Step 4 is central: when oxygenation worsens rather than improves after a cautious PEEP increase, a pressure-dependent intracardiac shunt should be suspected, and returning to a lower PEEP level may serve as both a diagnostic and a therapeutic maneuver. PEEP levels and increments should be individualized rather than applied uniformly. Abbreviations as in Table 2.

Beyond the intraoperative event, the incidental finding of a PFO raised the question of long-term management. In this patient, conservative management without PFO closure or antiplatelet therapy was recommended. Current evidence supporting percutaneous PFO closure derives chiefly from randomized trials in patients with PFO-associated cryptogenic stroke, in whom closure reduced recurrent stroke compared with medical therapy (12, 13); the expected benefit is greater when the PFO is more likely to be causally related to the stroke, as estimated by tools such as the Risk of Paradoxical Embolism (RoPE) score (14). Our patient had no history of cryptogenic stroke, transient ischemic attack, paradoxical embolism, or decompression illness, and the shunt was small and predominantly left-to-right at rest. He therefore did not meet established indications for closure, and there was no separate indication for antiplatelet or anticoagulant therapy. The clinical significance of his PFO was confined to a specific, modifiable physiological circumstance—high intrathoracic pressure during OLV—rather than an ongoing thromboembolic risk. The appropriate response was therefore to document the finding, counsel the patient, and inform future anesthetic management, rather than to pursue structural intervention (1, 6, 8).

This case report has several limitations. First, the absence of invasive hemodynamic monitoring (e.g., direct right atrial pressure measurement) prevented real-time quantification of intracardiac pressures during PEEP titration, which would have allowed more direct assessment of the proposed pressure-gradient reversal. Second, intraoperative TTE did not demonstrate definitive shunt flow, likely owing to suboptimal acoustic windows in the lateral decubitus position, the absence of agitated saline contrast, and time pressure during an evolving crisis; the intraoperative diagnosis therefore rested primarily on the temporal relationship between PEEP changes and oxygenation, supported by postoperative studies. Third, arterial blood gas analysis was not obtained at the nadir of desaturation, so the severity of hypoxemia could not be quantified (e.g., PaO_2_/FiO_2_ ratio). Fourth, preoperative PFO screening was not performed; routine screening is not generally performed in asymptomatic patients, yet this case illustrates a context in which an occult PFO can have important hemodynamic consequences. Fifth, the single-case design limits generalizability, and the true incidence of PEEP-associated paradoxical hypoxemia during OLV remains unknown. Finally, alternative ventilation strategies (e.g., CPAP to the non-ventilated lung) were not systematically tested during the crisis.

Within these constraints, the reproducible inverse relationship between PEEP and oxygenation, together with multimodal postoperative characterization of a PFO capable of right-to-left shunting, supports the proposed mechanism. When hypoxemia paradoxically worsens with PEEP escalation during OLV, clinicians should consider an occult intracardiac shunt and recognize that reduction of PEEP may serve as both a diagnostic probe and an effective therapeutic intervention (7, 8, 10).

## Conclusion

This case illustrates that PEEP, although a cornerstone of managing hypoxemia during one-lung ventilation, can paradoxically worsen oxygenation in patients with an occult patent foramen ovale, most likely through a pressure-dependent reversal of the interatrial gradient that may permit right-to-left shunting. The key clinical signal is counterintuitive: oxygenation that deteriorates rather than improves after PEEP escalation, despite stable hemodynamics and adequate ventilator mechanics, should prompt consideration of an intracardiac shunt. In this setting, reducing PEEP may act as both a diagnostic probe and a therapeutic maneuver, with rapid recovery supporting the suspicion of a pressure-dependent shunt. Given the high population prevalence of PFO (approximately 25%–30%), anesthesiologists should maintain awareness of this mechanism when standard recruitment strategies fail or worsen oxygenation during thoracic surgery. Recognizing this paradoxical response, rather than reflexively escalating airway pressure, may rapidly reverse refractory or unexplained hypoxemia.

## Data Availability

The datasets presented in this study can be found in online repositories. The names of the repository/repositories and accession number(s) can be found in the article/Supplementary Material.

## References

[1] Young CC Harris EM Vacchiano C Bodnar S Bukowy B Elliott RRD. Lung-protective ventilation for the surgical patient: international expert panel-based consensus recommendations. Br J Anaesth. (2019) 123:898–913. 10.1016/j.bja.2019.08.017 31587835

[2] Deng QW Tan WC Zhao BC Wen SH Shen JT Xu M. Intraoperative ventilation strategies to prevent postoperative pulmonary complications: a network meta-analysis of randomised controlled trials. Br J Anaesth. (2020) 124:324–35. 10.1016/j.bja.2019.10.024 32007240

[3] Hagen PT Scholz DG Edwards WD. Incidence and size of patent foramen ovale during the first 10 decades of life: an autopsy study of 965 normal hearts. Mayo Clin Proc. (1984) 59:17–20. 10.1016/S0025-6196(12)60336-X 6694427

[4] Tavazzi G Pozzi M Via G Mongodi S Braschi A Mojoli F. Weaning failure for disproportionate hypoxemia caused by paradoxical response to positive end-expiratory pressure in a patient with patent foramen ovale. Am J Respir Crit Care Med. (2016) 193:e1–2. 10.1164/rccm.201505-0967IM 26308849

[5] Palazzo P Heldner MR Nasr N Alexandrov AV. Transcranial Doppler with microbubbles: screening test to detect and grade right-to-left shunt after an ischemic stroke: a literature review. Stroke. (2024) 55:2932–41. 10.1161/STROKEAHA.124.046907 39268611

[6] Park M Yoon S Nam JS Ahn HJ Kim H Kim HJ. Driving pressure-guided ventilation and postoperative pulmonary complications in thoracic surgery: a multicentre randomised clinical trial. Br J Anaesth. (2023) 130:e106–18. 10.1016/j.bja.2022.06.037 35995638

[7] Pristipino C Sievert H D'Ascenzo F Mas JL Meier B Scacciatella P. European Position paper on the management of patients with patent foramen ovale. General approach and left circulation thromboembolism. Eur Heart J. (2019) 40:3182–95. 10.1093/eurheartj/ehy649 30358849

[8] Cappio Borlino S Hagry J Lai C Rocca E Fouqué G Rosalba D. The effect of positive end-expiratory pressure on pulmonary vascular resistance depends on lung recruitability in patients with acute respiratory distress syndrome. Am J Respir Crit Care Med. (2024) 210:900–7. 10.1164/rccm.202402-0383OC 38924520

[9] Di Cristo A Segreti A Tetaj N Crispino SP Guerra E Stirpe E. Hemodynamic effects of positive airway pressure: a cardiologist’s overview. J Cardiovasc Dev Dis. (2025) 12(3):97. 10.3390/jcdd12030097 40137095 PMC11942660

[10] Katsanos AH Psaltopoulou T Sergentanis TN Frogoudaki A Vrettou AR Ikonomidis I. Transcranial Doppler versus transthoracic echocardiography for the detection of patent foramen ovale in patients with cryptogenic cerebral ischemia: a systematic review and diagnostic test accuracy meta-analysis. Ann Neurol. (2016) 79:625–35. 10.1002/ana.24609 26833864

[11] Peel JK Funk DJ Slinger P Srinathan S Kidane B. Positive end-expiratory pressure and recruitment maneuvers during one-lung ventilation: a systematic review and meta-analysis. J Thorac Cardiovasc Surg. (2020) 160:1112–22.e3. 10.1016/j.jtcvs.2020.02.077 32276803

[12] Mas JL Derumeaux G Guillon B Massardier E Hosseini H Mechtouff L. Patent foramen ovale closure or anticoagulation vs. antiplatelets after stroke. N Engl J Med. (2017) 377:1011–21. 10.1056/NEJMoa1705915 28902593

[13] Saver JL Carroll JD Thaler DE Smalling RW MacDonald LA Marks DS. Long-term outcomes of patent foramen ovale closure or medical therapy after stroke. N Engl J Med. (2017) 377:1022–32. 10.1056/NEJMoa1610057 28902590

[14] Kent DM Ruthazer R Weimar C Mas JL Serena J Homma S. An index to identify stroke-related vs incidental patent foramen ovale in cryptogenic stroke. Neurology. (2013) 81:619–25. 10.1212/WNL.0b013e3182a08d59 23864310 PMC3775694

